# Mechanistic interaction studies of synthesized ZIF-8 nanoparticles with bovine serum albumin using spectroscopic and molecular docking approaches

**DOI:** 10.1038/s41598-022-14630-y

**Published:** 2022-06-20

**Authors:** Ashi Mittal, Sona Gandhi, Indrajit Roy

**Affiliations:** 1 Department of Chemistry, University of Delhi, Delhi, 110007 India; 2 Department of Chemistry, Galgotias University, Greater Noida, 203201 India

**Keywords:** Chemistry, Nanoscience and technology

## Abstract

Numerous studies have shown that nanosized zeolitic imidazolate framework particles (ZIF-8 NPs) serve as promising vehicles for pH-responsive drug delivery. An understanding of their interaction with serum proteins present in physiological systems will thus be of critical importance. In this work, monodisperse ZIF-8 NPs with an average size of 60 nm were synthesized at room temperature and characterized for their various physicochemical properties. Bovine serum albumin (BSA) was used as model serum protein for various interaction studies with ZIF-8 NPs. Spectroscopic techniques such as UV–visible and fluorescence spectroscopy indicated the formation of a ground-state complex with a binding constant of the order 10^3^ M^−1^ and a single binding site. Steady-state and time-resolved fluorescence spectroscopy confirmed the mechanism of quenching to be static. Conformational changes in the secondary structure of BSA were observed using CD and FT-IR spectroscopies. Binding sites were explored using molecular docking studies.

## Introduction

Metal–organic frameworks (MOFs) or porous coordination polymers (PCPs) are a class of coordination compounds in which metal ion vertices are interconnected by organic linkers to give highly porous structures with large internal surface area^1^. Zeolitic imidazolate frameworks (ZIFs) are a subclass of MOFs, structurally similar to conventional aluminosilicate zeolites, made up of M–Im–M type linkages (M = Zn or Co, Im = Imidazolate)^2^. One of the most studied ZIFs is ZIF-8, consisting of zinc metal ions and 2-methyl imidazolate (2-mIm) linkers. Both the constituents are found in physiological systems, zinc being the second most abundant transition element found in biological systems and the imidazole group an integral part of the amino acid histidine, making ZIF-8 highly biocompatible^3^. Nanosized ZIF-8 particles (ZIF-8 NPs) have been reported as efficient drug delivery systems owing to their properties such as (1) high surface area (BET: 1630 m^2^ g^−1^) and large pore size (11.6 Å), (2) high chemical and thermal stability, (3) high drug loading capacity and small window size (3.4 Å) preventing premature release of drug, (4) good dispersibility, and (5) pH-responsive drug release^4–7^. The protonation of the imidazolate group at pH 5.0–6.0 results in dissociation of the bond between zinc and imidazolate, thereby disassembling the nanostructure, which is ideal for drug release at tumor sites where the microenvironment is more acidic (pH 5.0–6.8) than in blood circulation and healthy tissues^8^.

Once nanoparticles are delivered within the body, they physically interact with biological macromolecules, particularly serum proteins present in blood and other body fluids^9^. Serum albumins account for almost 55% of the serum proteins present in the blood plasma^10^. Being naturally abundant in the blood, serum albumins are responsible for major functions such as maintenance of blood pH and osmotic pressure, and transportation of various endogenous-exogenous substances owing to their flexibility and ability to bind both hydrophilic and hydrophobic groups^11,12^. Bovine serum albumin (BSA) is one of the most studied serum albumins. It is a globular-protein and considered to be homologous to human serum albumin (HSA) with high sequence identity (> 70%)^13^. It is an inexpensive analog of HSA and more easily available. BSA consists of 583 amino acid residues divided amongst three structurally homologous domains (I, II, III) which are further divided into sub-domains^14^. There are two tryptophan residues (Trp134, Trp213) present, imparting BSA with intrinsic fluorescence^15^. Interactions between BSA and various noble metal NPs (such as Au, Ag)^16,17^, metal oxide NPs (such as ZnO, TiO_2_, CuO, iron oxide)^18–21^, ferrite NPs (such as manganese and cobalt ferrite)^22,23^, quantum dots^24,25^, etc., have been investigated. As per our knowledge, ZIF-8 NPs till now have not been systematically explored for their possible interaction with proteins, particularly BSA. An insight into this would be beneficial for future biomedical applications of these NPs.

In this work, we first synthesized ZIF-8 NPs and determined their various physicochemical properties. To investigate the interactions with BSA, we opted for a multi-spectroscopic and molecular docking approach. We employed UV–visible spectroscopy, along with steady-state and time-resolved fluorescence spectroscopies to determine binding constants between NPs and the protein. Circular Dichroism (CD) spectroscopy and Fourier-Transform Infrared (FT-IR) spectroscopy were employed to study conformational changes in the secondary structure of the protein upon binding. Molecular docking was performed to determine the binding sites and specific molecular interactions present between the NPs and different binding sites of BSA.

## Results and discussion

### Characterization of ZIF-8 NPs

**Figure 1:**
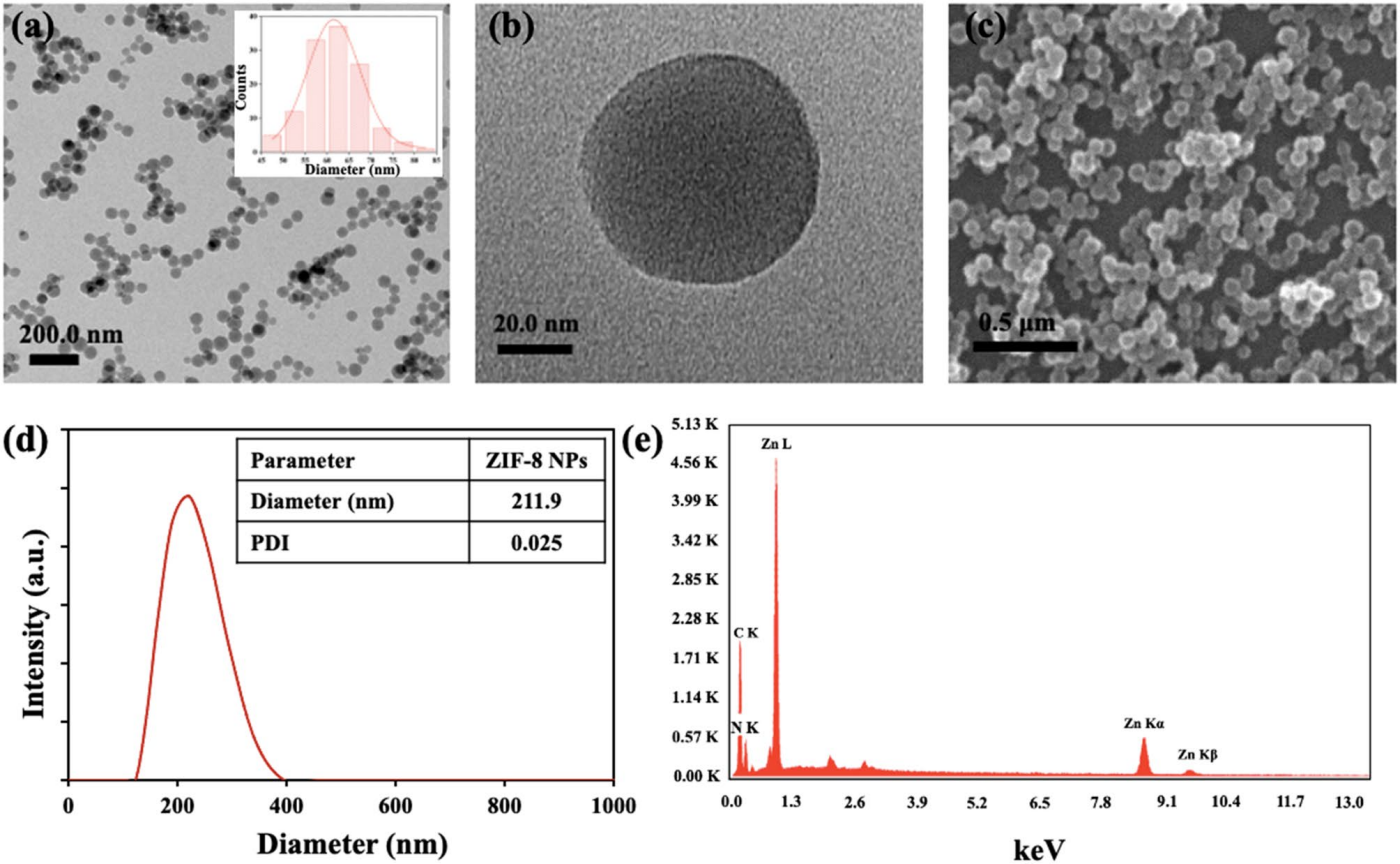
(**a**,**b**) TEM images of ZIF-8 NPs at a scale of (**a**) 200.0 nm and (**b**) 20.0 nm. (**c**) SEM image of ZIF-8 NPs. (**d**) DLS plot. (**e**) EDS plot confirming the elemental composition of ZIF-8 NPs.

Both TEM and SEM images of the as-synthesized ZIF-8 nanoparticles indicated the presence of monodispersed spherical particles with an average diameter of 60 nm (Fig. 1a–c). The synthesis conditions and time dictate the morphology of the nanoparticles. In our case, spherical nanoparticles were obtained as a result of a less reaction time of 5 min, whereas several other studies have reported rhombic dodecahedral shaped particles involving larger reaction time (12 h or more)^26,27^. The hydrodynamic diameter of the particles using DLS measurements was found to be 211.9 nm with a low polydispersity index (PDI) of 0.025, confirming formation of uniform particles (Fig. 1d). Since the hydrodynamic size is estimated by considering the hydration layer present on the particle surface, it is greater than the average size obtained from TEM analysis. Elemental analysis using EDS confirmed the presence of Zn, C, and N (Fig. 1e). The surface charge (zeta potential), measured at pH 7.0 (neutral) and 5.8 (weakly acidic), were + 21.1 mV and + 22.9 mV, respectively. The positive zeta-potential values can be attributed to the presence of excess zinc ions on the particle surface.

**Figure 2:**
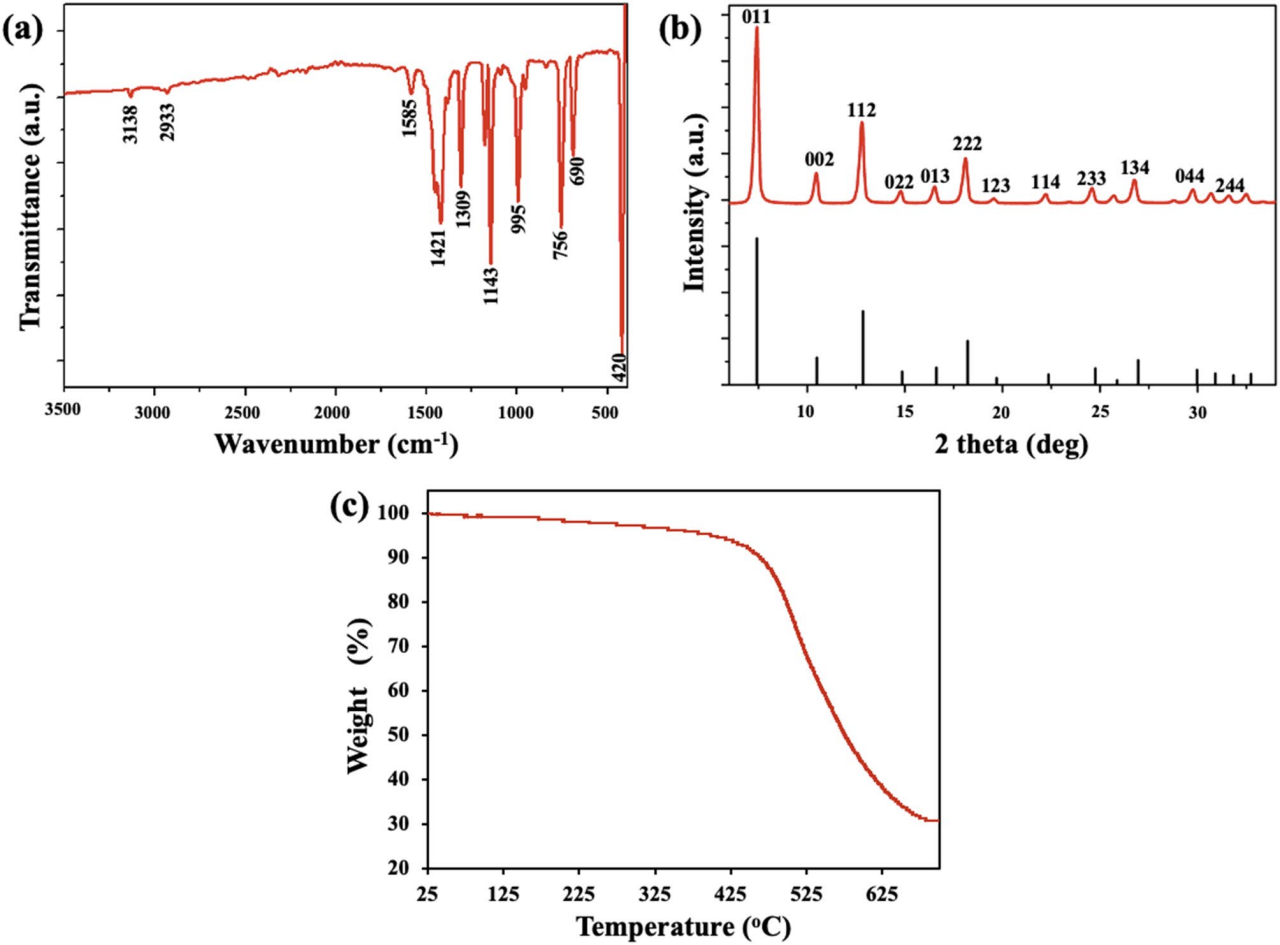
(**a**) FT-IR spectrum of ZIF-8 NPs. (**b**) Powder XRD pattern of ZIF-8 NPs (red) and simulated XRD pattern of ZIF-8 (black, ICDD No.: 00-062-1030). (**c**) TGA plot.

The FT-IR spectrum confirmed the successful synthesis of ZIF-8 NPs (Fig. 2a). The weak bands at 3138 and 2933 cm^−1^ are assigned to aromatic and aliphatic C–H stretch, respectively, of the imidazole group. The peak at 1585 cm^−1^ is attributed to C=N stretching and the intricated bands between 1350 and 1500 cm^−1^ are due to imidazole ring stretching. Bands in the region 900–1350 cm^−1^ and those below 850 cm^−1^ are associated with in-plane and out-of-plane bending of the imidazole ring, respectively. A strong band at 420 cm^−1^ is linked to Zn–N stretching^28^. Crystallinity and sodalite topology of ZIF-8 NPs was confirmed by XRD pattern (Fig. 2b). Comparison to simulated XRD pattern indicated the formation of pure-phase nanoparticles^29^. From the TGA plot (Fig. 2c), it was observed that there was a very small percentage of weight loss up to about 400 °C, indicating high thermal stability of as-formed nanoparticles. Beyond 400 °C, the weight loss is due to the decomposition of the 2-methylimidazole molecules^27^.

### Binding studies between ZIF-8 NPs and BSA

#### UV–visible spectroscopy

**Figure 3:**
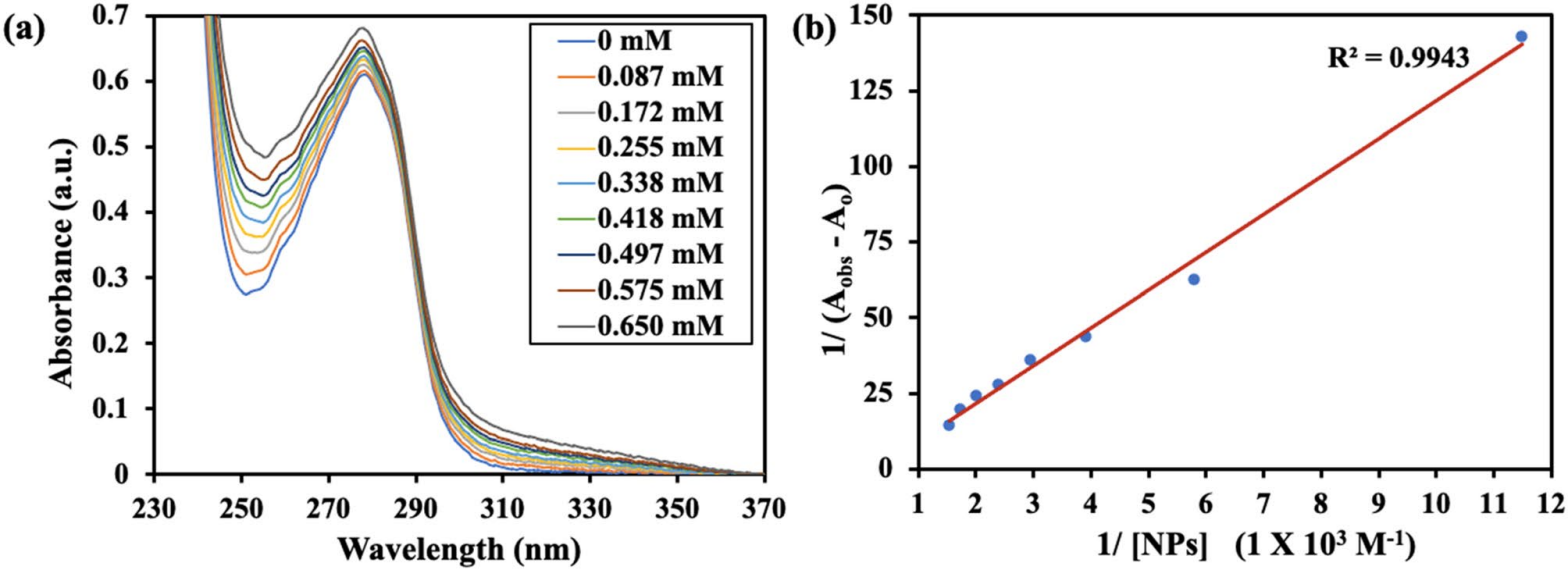
(**a**) Absorption spectra of BSA showing increase in absorbance values with increase in concentration of ZIF-8 NPs (in mM). (**b**) The plot of 1/(A_obs_ − A_o_) vs. 1/[ZIF-8 NPs] (Benesi–Hildebrand plot).

UV–visible spectroscopy is a powerful tool to study changes in protein conformation upon binding with a ligand. BSA shows two absorption peaks, a stronger peak at 210 nm reflecting framework conformation and another peak at 278 nm due to π → π* transition of aromatic amino acid residues (mainly Tyr, Trp)^30^. These absorption values may shift in magnitude and direction upon transfer of the chromophore to a different micro-environment, which tends to alter as a result of a change in protein conformation. The changes in the absorbance spectrum at 278 nm are generally observed for studying the binding interaction of proteins with ligands. It was observed, that on increasing the concentration of added ZIF-8 NPs to BSA, absorbance values at 278 nm increased, confirming the formation of a ground-state complex (Fig. 3a). Non-bonding interactions between a protein and a ligand can be quantitatively estimated using the Benesi–Hildebrand equation^31,32^. We used this equation to find the binding constant between BSA and ZIF-8 NPs.

$$ \frac{1}{{({\text{A}}_{{{{\rm obs}}}} - {\text{A}}_{{{\rm o}}} )}} = \frac{1}{{( {\text{A}}_{{{\rm c}}} - {\text{A}}_{{{\rm o}}} )}} + \frac{1}{{{\text{k}}_{{{{\rm app}}}} \left( {{\text{A}}_{{{\rm c}}} - {\text{A}}_{{{\rm o}}} } \right)[{\text{ZIF-8 NPs}}]}} $$

Here, A_obs_ and A_o_ denote the absorbance values at 278 nm in the presence and absence of ZIF-8 NPs, respectively. A_c_ is the maximum absorbance value in presence of nanoparticles and k_app_ is apparent binding constant. The corresponding plot gave binding constant value of 0.28 × 10^3^ M^−1^ (Fig. 3b). These results were suggestive of changes in protein conformation due to the formation of a stable complex between ZIF-8 NPs and BSA.

#### Steady-state fluorescence spectroscopy

**Figure 4:**
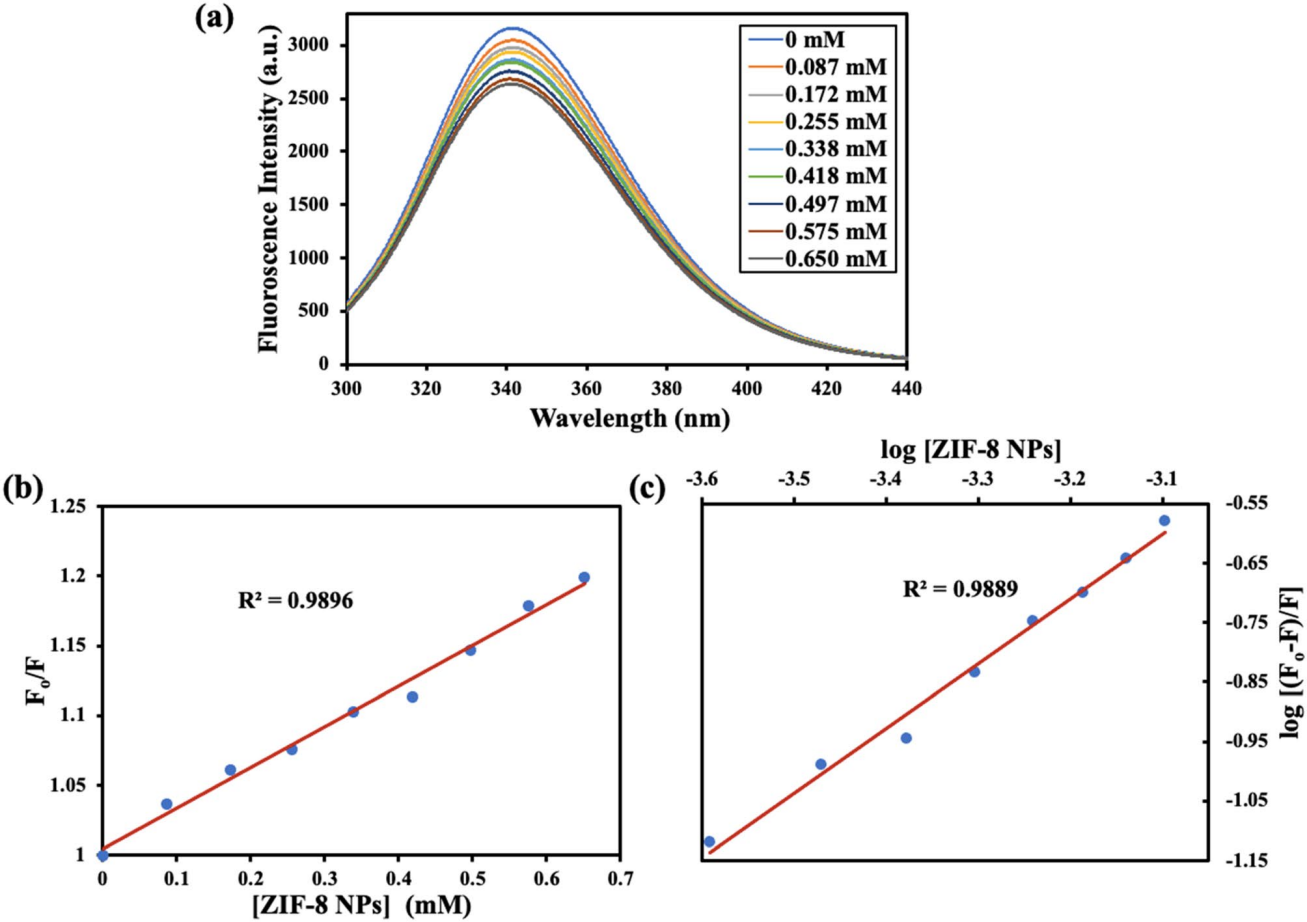
(**a**) Steady-state fluorescence spectra of BSA showing quenching of fluorescence with increasing concentration of ZIF-8 NPs. (**b**) The plot of F_o_/F versus [ZIF-8 NPs] (Stern–Volmer plot). (**c**) The plot of log [(F_o_ − F)/F] vs. log [ZIF-8 NPs] (double-logarithmic plot).

Various molecular interactions such as molecular-rearrangements, energy transfer, excited-state reactions, collision among molecules, and formation of ground-state complex may result in a decrease in fluorescence intensity of a fluorophore^33^. There are three intrinsic fluorophores present in BSA: phenylalanine, tyrosine, and tryptophan. Due to the low fluorescence quantum yield of phenylalanine, and very high quenching of tyrosine fluorescence in presence of an amino or carboxyl group, tryptophan is considered as the prime contributor towards intrinsic fluorescence of BSA^34^. The fluorescence intensity of tryptophan residues present in BSA is highly sensitive to a change in their micro-environment (hydrophobicity and polarity), and therefore can be used as an endogenous probe to monitor binding interactions. Analysis of fluorescence quenching of BSA in presence of ZIF-8 NPs showed a decrease in fluorescence intensity on increasing the concentration of nanoparticles (Fig. 4a). This fluorescence quenching can be a result of collisions between the fluorophore and the nanoparticles/quencher (dynamic quenching) or due to formation of a ground-state complex (static quenching). To verify the mechanism of quenching, we used the well-known Stern–Volmer equation^35^.

$$ \frac{{{\text{F}}_{{{\rm o}}} }}{{{\rm F}}} = {\text{K}}_{{{{\rm sv}}}} \left[ {\text{Q}} \right] + 1;\quad \left( {{\text{K}}_{{{{\rm sv}}}} = {\text{K}}_{{{\rm q}}} {\text{t}}_{{{\rm o}}} } \right) $$

Here, F_o_ and F represent BSA fluorescence intensity in the absence and presence of quencher, Q (ZIF-8 NPs), respectively. K_sv_ is the Stern–Volmer quenching constant, K_q_ the quenching constant, and τ_o_ the average fluorescence lifetime (10^−8^ s for BSA). The plot between F_o_/F and concentration of ZIF-8 NPs (Fig. 4b) gave a value of 3.13 × 10^−2^ M^−1^ for K_sv_, and a corresponding value of 3.13 × 10^10^ M^−1^ s^−1^ for K_q_. The observed value for K_q_ is higher than the maximum value of the collision quenching constant (2.0 × 10^10^ M^−1^ s^−1^), confirming static quenching due to the formation of a ground-state complex between BSA and ZIF-8 NPs. The ground-state complex formed is stabilized mainly by one or more non-covalent binding forces, such as van der waals forces, hydrogen bonding, hydrophobic interactions, and electrostatic interactions^36^.

The static binding constant (K_b_) and the number of binding sites (n) were determined using the double-logarithmic plot (Fig. 4c) based on modified Stern–Volmer equation^37^.

$$ \log \left( {\frac{{{\text{F}}_{{{\rm o}}} - {{\rm F}}}}{{\text{F}}}} \right) = {\text{n}}\log \left[ {\text{Q}} \right] + \log {\text{K}}_{{{\rm b}}} $$

The value for the number of binding sites from the plot came out to be 1.09, indicating a single binding site present in BSA for nanoparticles. The binding constant between ZIF-8 NPs and BSA was found to be 0.59 × 10^3^ M^−1^. The results from fluorescence quenching measurements are therefore in agreement with our observations in changes in UV–visible spectra of protein upon addition of nanoparticles. Dynamic quenching does not affect the absorption spectrum of the protein; however, formation of ground-state complex due to static quenching is reflected by changes in both fluorescence and UV–visible absorption spectra.

#### Time-resolved fluorescence spectroscopy

**Figure 5:**
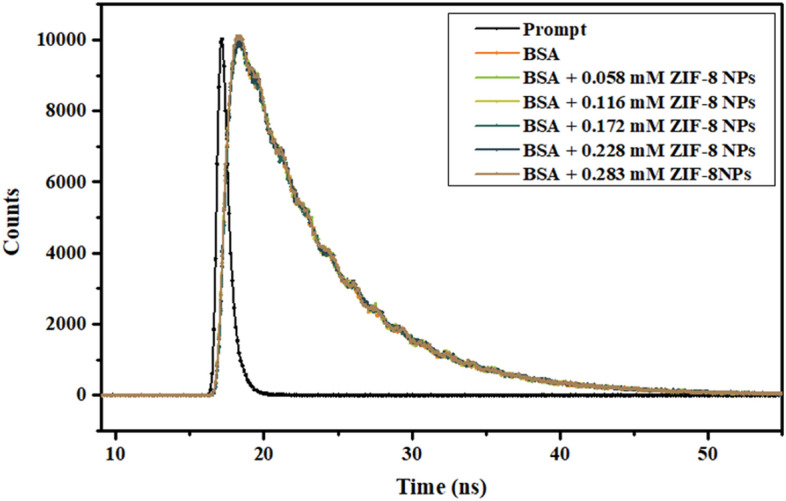
Time-resolved fluorescence study of BSA in presence of different concentration of ZIF-8 NPs.

Measurement of decay in fluorescence lifetimes can also help in ascertaining the nature of fluorescence quenching. In the process of dynamic quenching, a decrease in average-lifetime value of fluorophore is observed. Whereas, in case of static quenching no change in lifetime value of fluorophore (un-complexed) is observed due to formation of non-fluorescent complex^38^. The two tryptophan residues present in BSA are in different local environments; Trp134 is on the surface and Trp213 is deeply buried^39^. BSA did not show any considerable change in fluorescence with time with the addition of ZIF-8 NPs (Fig. 5). Two exponentials, τ_1_ and τ_2_, were used for fluorescence decay curve fitting. Average-lifetime values (τ_av_) were obtained from lifetime values (τ_1_, τ_2_), pre-exponential values (a_1_, a_2_) and normalized pre-exponential values (α_1_, α_2_) (Table 1)^40^.

$$ \uptau _{{\rm av}}  = \sum\limits_{{\rm i}}^{{\rm n}} {\upalpha _{{\rm i}} \uptau _{{\rm i}} } ;\quad {\text{where}}\quad \upalpha _{{\rm i}}  = {\text{a}}_{{\rm i}} /\sum\limits_{{\rm i}}^{{\rm n}} {{\text{a}}_{{\rm i}}}  ,\quad {\text{and}}\quad \sum\limits_{{\rm i}}^{{\rm n}} {\upalpha _{{\rm i}} }  = 1 $$

**Table 1:** Fluorescence lifetime decay parameters for BSA in presence of different concentrations of ZIF-8 NPs.

[ZIF-8 NPs] (mM)	τ_1_ (ns)	τ_2_ (ns)	a_1_	a_2_	τ_av_ (ns)
0	3.80	6.68	0.035	0.105	5.96
0.058	3.73	6.66	0.031	0.109	6.01
0.116	3.57	6.63	0.028	0.112	6.02
0.172	3.34	6.67	0.028	0.111	6.00
0.228	3.41	6.64	0.026	0.114	6.04
0.283	3.67	6.73	0.036	0.106	5.95

A considerable difference between values of τ_1_ and τ_2_ confirmed presence of two tryptophan residues in different microenvironments. Also, fairly constant average-lifetime values in the presence and absence of ZIF-8 NPs confirmed static quenching as the formation of a complex does not alter the lifetime values^41^.

#### Circular dichroism (CD) spectroscopy

**Figure 6:**
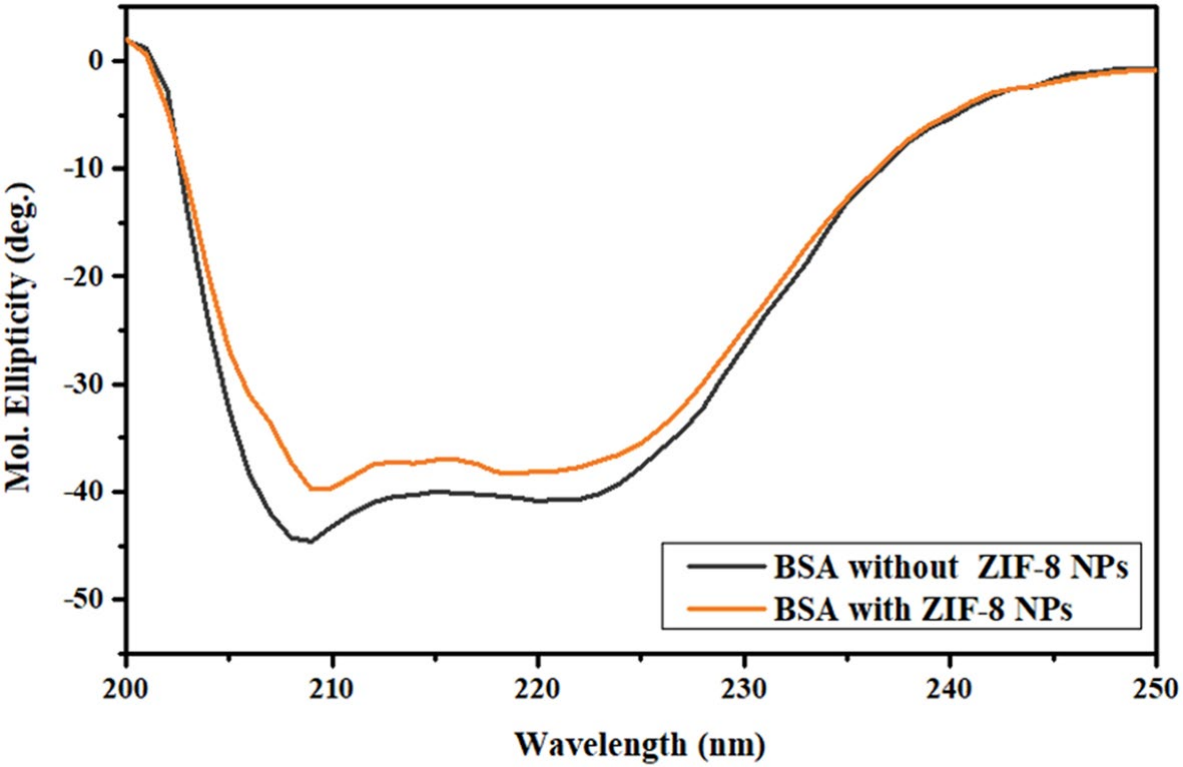
Far UV-CD spectra of BSA in presence and absence of ZIF-8 NPs.

CD spectroscopy in the far UV region can be utilized for studying changes in secondary structure of a protein. BSA is an α-helix rich protein. The characteristic CD spectrum of BSA shows two negative bands at 208 and 222 nm corresponding to π → π* and n → π* electronic transitions, respectively for α-helix secondary structure^42^. Any change in position and intensity of these bands indicates change in conformation of the protein upon binding. On addition of ZIF-8 NPs, a decrease in magnitude of molar ellipticity values with a slight shift in peak position was observed (Fig. 6). Mean residual ellipticity values (MRE) (deg cm^2^ dmol^−1^) at 208 nm were obtained from observed molar ellipticity values (deg), which were then used to determine α-helix content (%) in BSA, with and without added NPs^43^.

$$ \begin{aligned} & {\text{MRE}} = \frac{{{\text{observed molar ellipticity}}\;(\deg )}}{{{\text{C}}_{{{\rm p}}} {\text{n }}l \times 10}} \\ & \upalpha{\text{-helix}}\;\left( \% \right) \, = \frac{{ - {\text{MRE}}_{208} - 4000}}{33000 - 4000} \\ \end{aligned} $$

Here, C_p_ denotes molar concentration of BSA, *l* is cuvette path length (1 cm), and n is amino acid residues present (583 for BSA). A decrease in α-helix content was observed in presence of NPs (Table 2). This decrease was indicative of change in protein conformation due to insertion of nanoparticle into the binding site of protein. The hydrogen bonding network present in α-helical structure of the protein is perturbed due to interaction of particle with amino acid residues present in the binding cleft^44^. The characteristics peaks corresponding to α-helix secondary structures are retained, however, lower in intensity, suggesting some unfolding of the protein without any destructive conformation changes. These results are consistent with the results of fluorescence and UV–visible spectroscopy.

**Table 2:** Percentage α-helix content of BSA in presence and absence of ZIF-8 NPs.

[ZIF-8 NPs] (µM)	% α-helix
0	38.6
67.6	31.1

#### Fourier-transform infrared (FT-IR) spectroscopy

**Figure 7:**
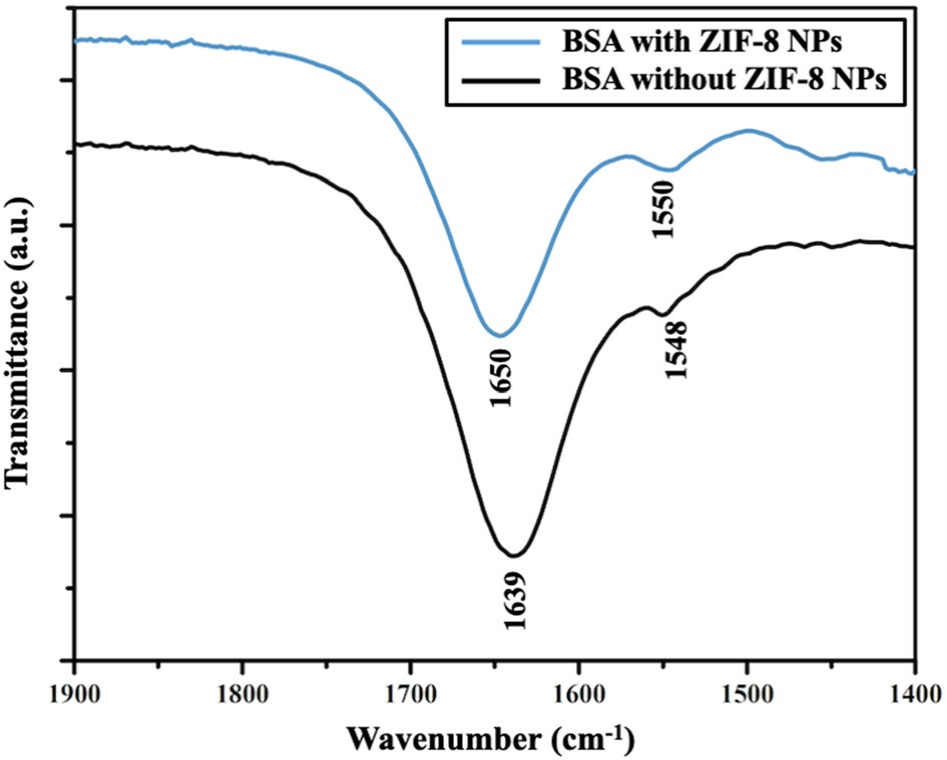
FT-IR spectra of BSA showing changes in amide-I and amide-II bands upon interaction with ZIF-8 NPs.

Characteristic amide-I (1600–1700 cm^−1^) and amide-II (1500–1600 cm^−1^) bands in the mid-IR region are associated with secondary structure of a protein. Amide-I band is linked to C=O stretching, while amide-II band is mainly attributed to coupling between C–N stretching and N–H bending and less influenced by a change in the structure^45^. BSA showed a significant shift in position of amide-I band from 1639 to 1650 cm^−1^ and a very slight shift in position of amide-II band from 1548 to 1550 cm^−1^ upon interaction with ZIF-8 NPs in the FT-IR spectrum (Fig. 7). The significant shift in position of amide-I band due to stretching of C=O can be possibly explained on the basis of hard and soft acid base (HSAB) theory. This theory can help in predicting the selective interaction between metal ions and functional groups present at the binding sites of protein. Metal ions preferentially bind to protein sites having similar hardness or softness^46^. Zinc ion (Zn^2+^; present in ZIF-8 NPs) is a borderline acid and C=O group is a soft base. Their interaction will result in conformational changes in the secondary structure of the protein, which can be reflected in the changes in the position of amide bands of BSA upon addition of ZIF-8 NPs.

#### Molecular docking

**Figure 8:**
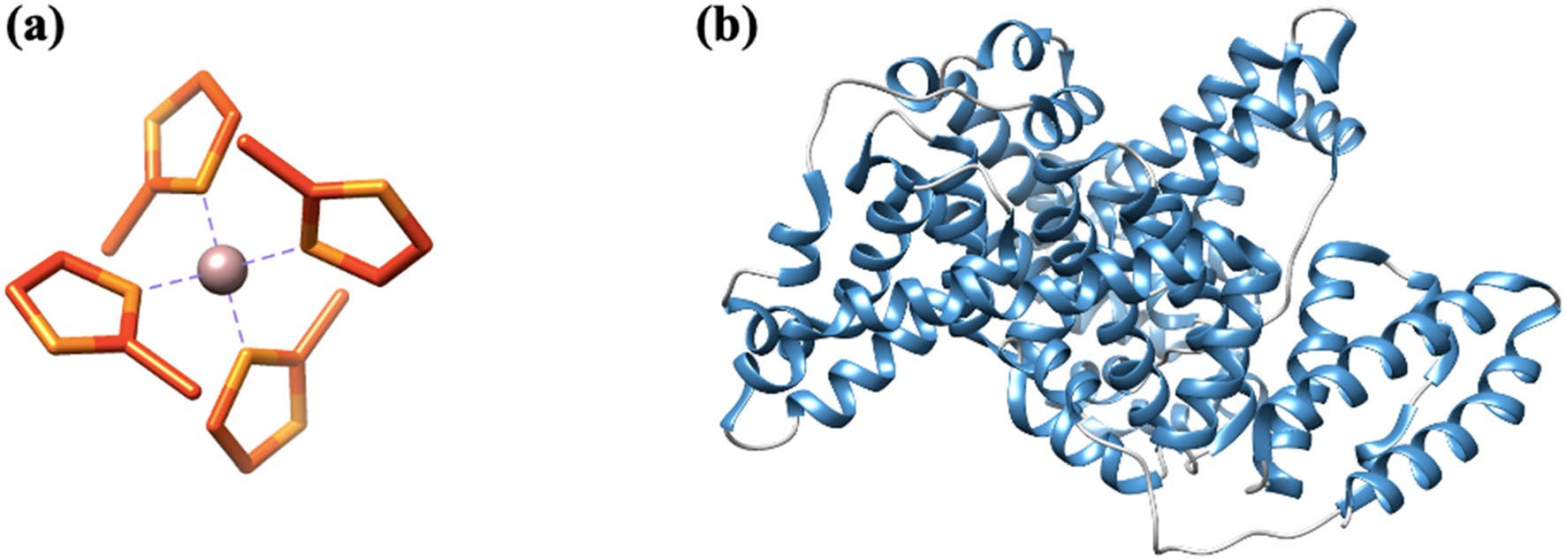
(**a**) Secondary building unit of ZIF-8 NPs, [Zn(2-mIm)_4_] (Zn: purple, C: orange, N: yellow). (**b**) Structure of BSA protein chain A (α-helix: blue, random coil: grey).

**Figure 9:**
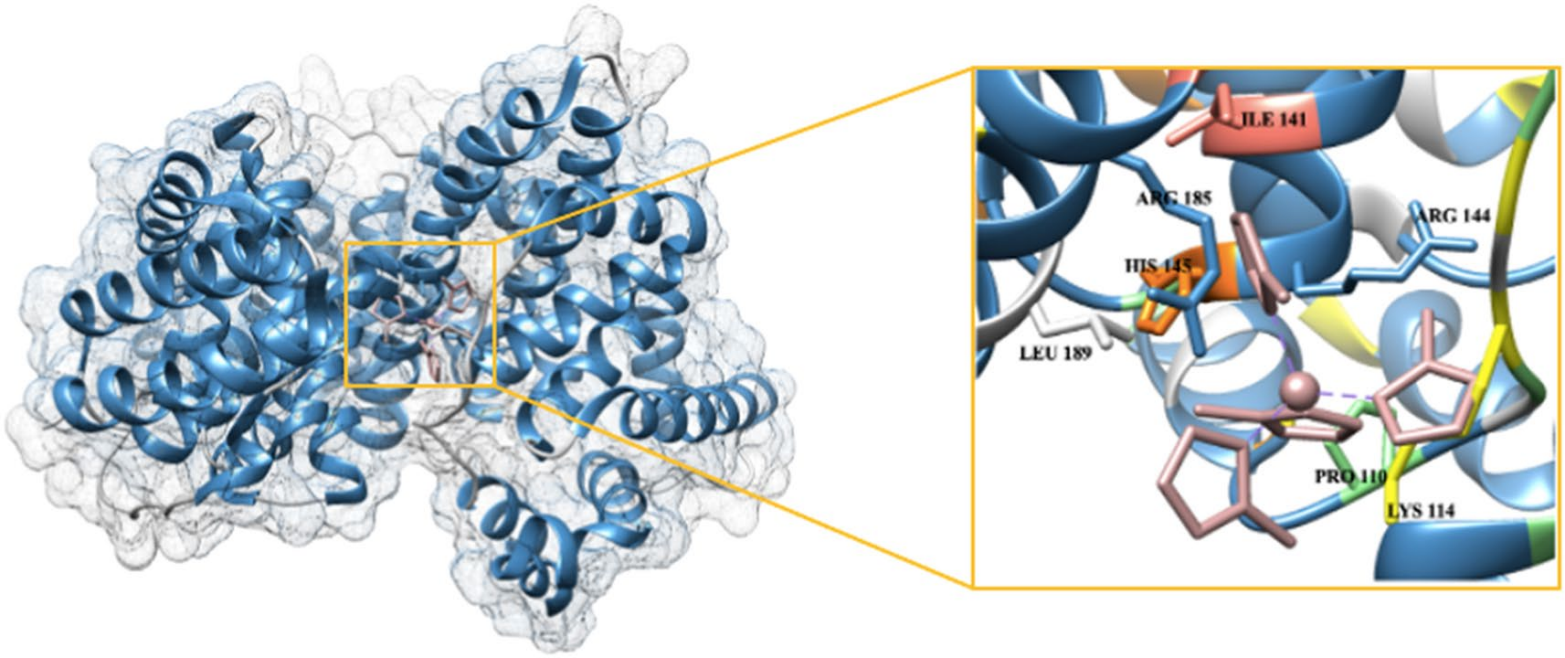
Surface view showing interaction between protein (blue) and ZIF-8 SBU (pink) with an enlarged image of the binding groove showing amino acid residues involved in molecular interactions.

The secondary building units (SBUs) present in MOFs are metal ion clusters joined to each other by organic linkers. These lock the position of metal ion into a fixed geometry, giving a rigid framework. The SBU of ZIF-8 (Fig. 8a), consisting of zinc ion tetrahedrally linked to four 2-methyl imidazole groups, was chosen for studying the interaction of ZIF-8 NPs with BSA. This is the basic unit for ZIF-8 NPs, as linking of these SBUs to one another results in formation of three-dimensional network of ZIF-8. Three-dimensional structure of BSA (Fig. 8b) shows presence of mainly α-helices. The complex of protein with ZIF-8 unit (Fig. 9) shows that ZIF-8 fits in the cavity of protein chain. The values for interface area and atomic contact energy were obtained to be 736.70 Å^2^ and 113.29 kcal mol^−1^, respectively, indicating strong binding. Seven amino acid residues were involved in binding (Lys114, Arg144, His145, Arg185, Leu189, Ile141, Pro110). The amino acid residues Leu 189, Ile141 and Pro110 have hydrophobic side chains; whereas Lys114, Arg144, Arg185 and His145 have positively charged groups. It can be inferred that the binding between ZIF-8 unit and BSA is mainly due to hydrophobic interactions, with some minor contribution from electrostatic interactions.

For molecular docking studies, we considered a single SBU of ZIF-8, instead of a full nanoparticle. We assumed that even if there are many SBUs in the ligand file, their binding with the protein will be at some specific region (binding cavity) only. Moreover, even after adding more repeating units, there will not be any significant change in the binding free energy; possibly due to the reason that the binding groove of the protein is already occupied with the single repeating unit of the nanoparticle. Although the role of nanoparticle morphology and the structural changes in protein upon binding cannot be deciphered by this docking approach, it gives vital information about the binding process. It confirms that the binding is taking place with appreciable interface area and atomic contact energy. It further reveals the possible binding site in the protein and the amino acids responsible for interaction. These findings from molecular docking validated the previously obtained results from spectroscopic techniques. The binding of ZIF-8 unit in the protein cavity will disrupt the α-helix secondary structure, which explains the observations made in the far-UV CD and FT-IR spectra of BSA. The resulting change in protein conformation will affect the micro-environment of the nearby tryptophan residues. Also, the presence of substantial binding between ZIF-8 SBU and BSA confirms that there is formation of a ground state-complex and is in accordance with the results obtained from UV–visible and fluorescence spectroscopic studies.

## Material and methods

### Materials

Zinc nitrate hexahydrate (Zn(NO_3_)_2_·6H_2_O, 99%) with CAS number 10196-18-6, bovine serum albumin (BSA, ≥ 98%) with CAS number 9048-46-8, and 1× phosphate buffered saline (PBS, pH = 7.4 ± 0.1) were obtained from Sisco Research Laboratories (SRL) Pvt. Ltd., India. 2-Methyl imidazole (C_4_H_6_N_2_, ≥ 99%) with CAS number 693-98-1, was procured from Central Drug House (CDH) (P) Ltd., India. Methanol (HPLC grade) was purchased from Spectrochem Pvt. Ltd., India. All chemicals were used as received, without any further purification.

### Synthesis of ZIF-8 NPs

Nanosized ZIF-8 particles were synthesized according to a previous report^47^. A solution containing 150 mg zinc nitrate hexahydrate dissolved in 7.15 mL of methanol was kept for magnetic stirring at room temperature. To this, a solution containing 330 mg 2-Methyl Imidazole dissolved in 7.15 mL methanol was added dropwise under constant stirring. After stirring for 5 min, the reaction solution became milky white indicating the formation of nanoparticles. The particles were separated by centrifuging at 8000 rpm and unreacted reagents were removed subsequently by washing with methanol. The particles were then dried for further analysis.

### Characterization of ZIF-8 NPs

The as-prepared ZIF-8 NPs were characterized for their physicochemical properties before carrying out interaction studies. The size and morphology of the particles were determined using transmission electron microscopy (TEM), scanning electron microscopy (SEM), and dynamic light scattering (DLS) measurements. A dilute sample of ZIF-8 NPs dispersed in methanol was drop casted on carbon-coated copper grids, followed by air-drying and analysis using TALOS TEM (Thermo Scientific) operating at 200 kV. Nanoparticles dispersed in methanol were drop casted on a glass slide and air-dried, and then SEM analysis was carried out using JEOL JSM-6610 operating at 20 kV. Nanoparticles were well dispersed in water and the Malvern Zetasizer instrument was used for DLS and zeta potential analysis. Elemental composition was verified by energy dispersive spectroscopy (EDS) using JEOL JSM-6610 instrument. Fourier-transform infrared (FT-IR) spectroscopy was used to analyze surface functionalities using Shimadzu IRAffinity-1S spectrophotometer. Shimadzu DTG-60 simultaneous DTA-TG apparatus was used for thermogravimetric analysis (TGA) of nanoparticles in nitrogen atmosphere. The crystallinity of particles was determined using the Rigaku Miniflex benchtop powder X-ray diffraction (XRD) instrument.

### Binding studies between ZIF-8 NPs and BSA

#### UV–visible spectroscopy

Stock solutions of BSA (15 μM, in PBS) and ZIF-8 NPs (2 mg/mL, 8.8 mM, in methanol) were prepared. UV–visible spectra were recorded using Lab India UV 3092 spectrophotometer in the wavelength range of 200–400 nm. The concentration of BSA was kept constant at 15 μM and the concentration of NPs was varied from 0 to 0.65 mM with a fixed incubation time of 1 min. Absorbance values at 278 nm were recorded and were used to determine the binding constant between the particles and protein by plotting the Benesi-Hildebrand graph.

#### Steady-state fluorescence spectroscopy

Fluorescence spectra were recorded using a Hitachi F-4700 fluorescence spectrophotometer by setting excitation wavelength at 278 nm for BSA and fluorescence intensity values were recorded at an emission wavelength of 340 nm. The concentration of BSA (in PBS) was kept constant at 15 μM and the concentration of NPs was varied from 0 to 0.65 mM during successive measurements with a fixed incubation time of 1 min. Emission values obtained were used to determine the nature of quenching by using the Stern–Volmer equation. The number of binding sites and binding constant was determined using a double-logarithmic plot.

#### Time-resolved fluorescence spectroscopy

The decay in fluorescence of BSA with time was observed using Horiba-Jobin Yvon spectrometer with nanoLED set at 280 nm providing excitation pulse of 1.2 ns and 1 MHz pulse repetition rate. All spectra were recorded at room temperature and decay in lifetime was measured using time-correlated single-photon counting, TCSPC technique. Prompt spectrum was recorded followed by lifetime measurements for protein by keeping the concentration of BSA constant (15 μM, in PBS) and varying the concentration of ZIF-8 NPs.

#### Circular dichroism (CD) spectroscopy

Conformational analysis was carried out by recording circular dichroism data for BSA (0.5 μM, in PBS), with and without NPs, using the Jasco J815 CD spectro-polarimeter. Quartz cuvette (path length, 1 cm) was used for all measurements, and nitrogen was fluxed continuously throughout the experiment. Baseline correction was done using PBS. An average of three scans was taken for each spectrum and recorded in the far UV region (190–250 nm) at 298 K with a response time of 1 s, scanning speed of 100 nm min^−1^, and bandwidth of 1 nm.

#### Fourier-transform infrared (FT-IR) spectroscopy

FT-IR spectra were recorded using Shimadzu IRAffinity-1S spectrophotometer in the spectral range of 1400–1800 cm^−1^. Spectra of BSA was recorded in presence and absence of ZIF-8 NPs. Concentration of BSA was kept constant at 1 mM (in PBS). For interaction study, concentration of NPs was taken as 5 mM and the sample was allowed to equilibrate for 1 h at room temperature.

#### Molecular docking

The crystallographic information file (.CIF) for ZIF-8 was obtained from structural database of CCDC (deposit number 864309). It was converted into .mol format using an open-source software, Open Babel. This file was then utilized to draw secondary building unit (SBU) of ZIF-8 in ChemDraw and saved in .mol format. Open Babel was again used to convert this file into .pdb format. The database of the Protein Data Bank was used for deriving the structure of BSA (ID-4O30) in .pdb format. BSA is made up of two identical chains. Chain A was selected for docking. The visualization software, UCSF Chimera-1.15 was used to view three-dimensional structure of the protein chain and the ZIF-8 SBU. The PatchDock molecular docking server, based on principles of shape-complementarity, was used^48^. The .pdb files of receptor (BSA) and ligand (ZIF-8) were uploaded with cluster RMSD value, 1.5 Å and protein-small ligand setting in complex type. The lowest energy docked complex was then analyzed for possible binding sites and visualized using UCSF Chimera.

## Conclusion

The increasing research on the use of nanoparticles for biomedical applications demands extensive investigation of the interactions of nanoparticles with the constituents present in blood and other body fluids. The binding of nanoparticles with serum albumins present in the blood is responsible for their in vivo transportation, absorption, and distribution, which ultimately affects their stability, therapeutic efficacy, and toxicity. In the present study, we have observed the formation of a 1:1 ground-state complex between the nanoparticles and the protein, with a binding constant of the order 10^3^ M^−1^. The static nature of the quenching mechanism also indicated binding of ZIF-8 NPs with the protein.

Binding of the common serum protein BSA on ZIF-8 surface can significantly alter the pharmacokinetics, biodistribution and degradation profile of the nanoparticle. In drug delivery, such interactions are often detrimental to the optimal performance of the nanoparticle. For example, increased size of protein-bound nanoparticles can lead to their enhanced phagocytic capture and degradation. In the case of ZIF-8 nanoparticles, such protein binding can hamper with their pH-controlled drug release property. Therefore, the surface of these nanoparticles should be extensively functionalized to reduce their hydrophobicity and/or cationic charge, so that non-specific protein binding is reduced. Further comprehensive studies will be useful in understanding the biological effects of these nanoparticles and their possible use as drug delivery vehicles for clinical studies in the future.

## Data Availability

The datasets used and/or analyzed during the current study available from the corresponding author on reasonable request.
